# Active contact proximity to the cerebellothalamic tract predicts initial therapeutic current requirement with DBS for ET: an application of 7T MRI

**DOI:** 10.3389/fneur.2023.1258895

**Published:** 2023-11-09

**Authors:** Salman S. Ikramuddin, Annemarie K. Brinda, Rebecca D. Butler, Meghan E. Hill, Rajiv Dharnipragada, Joshua E. Aman, Lauren E. Schrock, Scott E. Cooper, Tara Palnitkar, Rémi Patriat, Noam Harel, Jerrold L. Vitek, Matthew D. Johnson

**Affiliations:** ^1^Department of Neurology, University of Minnesota, Minneapolis, MN, United States; ^2^Department of Biomedical Engineering, University of Minnesota, Minneapolis, MN, United States; ^3^University of Minnesota Medical School, Minneapolis, MN, United States; ^4^CMRR, University of Minnesota, Minneapolis, MN, United States

**Keywords:** deep brain stimulation, essential tremor, cerebellothalamic tract, 7-T MRI, stimulation amplitude, diffusion tractography imaging

## Abstract

**Objective:**

To characterize how the proximity of deep brain stimulation (DBS) active contact locations relative to the cerebellothalamic tract (CTT) affect clinical outcomes in patients with essential tremor (ET).

**Background:**

DBS is an effective treatment for refractory ET. However, the role of the CTT in mediating the effect of DBS for ET is not well characterized. 7-Tesla (T) MRI-derived tractography provides a means to measure the distance between the active contact and the CTT more precisely.

**Methods:**

A retrospective review was conducted of 12 brain hemispheres in 7 patients at a single center who underwent 7T MRI prior to ventral intermediate nucleus (VIM) DBS lead placement for ET following failed medical management. 7T-derived diffusion tractography imaging was used to identify the CTT and was merged with the post-operative CT to calculate the Euclidean distance from the active contact to the CTT. We collected optimized stimulation parameters at initial programing, 1- and 2-year follow up, as well as a baseline and postoperative Fahn-Tolosa-Marin (FTM) scores.

**Results:**

The therapeutic DBS current mean (SD) across implants was 1.8 mA (1.8) at initial programming, 2.5 mA (0.6) at 1 year, and 2.9 mA (1.1) at 2-year follow up. Proximity of the clinically-optimized active contact to the CTT was 3.1 mm (1.2), which correlated with lower current requirements at the time of initial programming (R^2^ = 0.458, *p* = 0.009), but not at the 1- and 2-year follow up visits. Subjects achieved mean (SD) improvement in tremor control of 77.9% (14.5) at mean follow-up time of 22.2 (18.9) months. Active contact distance to the CTT did not predict post-operative tremor control at the time of the longer term clinical follow up (R^2^ = -0.073, *p* = 0.58).

**Conclusion:**

Active DBS contact proximity to the CTT was associated with lower therapeutic current requirement following DBS surgery for ET, but therapeutic current was increased over time. Distance to CTT did not predict the need for increased current over time, or longer term post-operative tremor control in this cohort. Further study is needed to characterize the role of the CTT in long-term DBS outcomes.

## 1. Introduction

Essential tremor (ET) is a common movement disorder with rates as high as 5.8% in the geriatric population (1). ET presents as a postural and kinetic tremor typically affecting the upper extremities, head, or voice, with a subset of patients suffering from gait ataxia (2). This disorder is slowly progressive and traditionally familial. Tremor can vary widely in severity and lead to significant disability (3–5). Medical management of ET is limited to pharmacotherapy and surgical treatments including deep brain stimulation (DBS).

While the ventral intermediate (VIM) nucleus of the thalamus is the established surgical target for DBS in treating ET (4), more recently, there has been an increased interest in nearby neuroanatomic regions that may be implicated in the therapeutic effect of DBS (6). This work has energized investigation into the role that lead location, specifically the position of the lead relative to local fiber pathways, plays in the therapeutic effect of deep brain stimulation for the treatment of ET.

One such pathway of interest is the cerebellothalamic tract (CTT), coursing from the deep cerebellar nuclei to the ventrolateral thalamus (7). Activation of cerebellothalamic pathways with DBS has been associated with tremor reduction (8), and robust tremor suppression can be achieved via MR-guided focused ultrasound of the CTT in patients with chronic therapy-resistant ET (9). Similarly, in a computational study utilizing directional DBS leads and multi-compartment neuron models of the thalamocortical, cerebellothalamic, and medial lemniscal pathways, tremor suppression was most closely associated with activation of the CTT (10, 11). Further study is needed to build upon these studies to better understand the role of the CTT pathway over time.

DBS outcomes can be influenced by stimulation parameters, microlesion effect, habituation to stimulation, disease progression, and electrode location (12). Directional DBS leads, when clinically optimized, were able to reduce the required therapeutic current by 31%, resulting in an increase in the therapeutic window (13). Similarly, when DBS targeting the posterior subthalamic area (PSA) was compared to targeting the VIM, lower therapeutic current strength was required to produce a superior clinical outcome (14). Recent advances in ultra-high field (7T) MRI-derived fiber tractography provide opportunities to reconstruct neural pathways of interests with greater spatial precision than previously possible (15, 16). These approaches enable more precisely quantifying the impact of lead location on DBS outcomes with a high degree of spatial precision.

In this study, we sought to better understand the predictors of DBS outcomes in ET patients by leveraging 7T MRI to reconstruct the patient specific CTT, define a centerline of the CTT, and calculate the proximity of this centerline to the active DBS contact during monopolar review. We then investigated the relationship between this distance and post-operative stimulation parameters testing the hypothesis that active contact proximity to the CTT predicts the therapeutic current requirement and post-operative tremor control following DBS for ET.

## 2. Materials and methods

### 2.1. Study participants and clinical assessments

We performed a retrospective review of 7 subjects (3 female, 4 male) and 12 brain hemispheres who underwent VIM DBS surgery for ET with pre-operative 7T MRI at the University of Minnesota (Table 1). The study was approved by the University of Minnesota Institutional Review Board and all subjects gave written, informed consent prior to participation and in accordance with the Declaration of Helsinki. Dates of surgery ranged from June 2014 to May 2019. All patients were defined as candidates for DBS based on failed pharmacologic management and a pre-operative assessment that included comprehensive multi-disciplinary review involving neurology, neurosurgery, and neuropsychology as well as nursing and bioethics when required. DBS lead implant locations were determined by intraoperative microelectrode recording and macro-stimulation following lead placement to determine the current required to induce a therapeutic effect relative to the current required to induce side effects (paresthesia, dysarthria). We included 5 subjects with bilateral DBS lead placements, and unilateral left in 2 subjects. This included 11 directional Abbott Infinity 6172ANS leads and 1 omnidirectional Medtronic 3,389 lead. Clinical variables were collected for all patients including demographics, disease duration, side effects, stimulation thresholds, and stimulation parameters (amplitude [mA], frequency [Hz], and pulse width [μs]) at the initial programming visit in addition to existing stimulation parameters at the 1-year and 2-year time points. In a single subject with a Medtronic DBS lead, current was converted from voltage to milliamps (mA) using the charted impedances from the clinical visits at the time of follow up. Electrode geometry was collected as follows: The anode and cathode were extracted from the chart, and the segment selected was also reported. Segments ranged in value from 1 to 4, with their directionality indicated by the letters A, B, or C. The initial programming visit represented the first clinical follow-up after DBS surgery. At this time, DBS leads were activated, and a monopolar review of each contact was performed with increasing stimulation amplitudes. During this time, attention was paid to the degree of tremor reduction and the development of persistent stimulation-induced side effects at each contact. At the conclusion of this visit, an electrode configuration optimizing tremor reduction, without inducing persistent clinical side effects, was chosen. Thresholds for persistent side effects were collected when the subject reported non-transient side effects. Tremor severity at baseline and post-operative follow-up was assessed via the Fahn-Tolosa-Marin (FTM) tremor assessment. The components of the FTM hemi-score, with a scale from 0 (normal) to 4 (severe), included the upper and lower extremity tremor severity, drawing assessment, handwriting task in the dominant hand, and a liquid pouring exercise. Pre-operative FTM hemi-scores, first available post-operative FTM hemi-scores, and off-stim FTM hemi-scores were collected from the patient record. ON- and OFF-stim scores were frequently available in the same visit. Post-operative clinical tremor control was defined as the difference between the post-operative and pre-operative FTM-hemi scores, as well as the percentage improvement in FTM score scaled to baseline.

**Table 1:** Summary of subjects.

Age	Sex	Electrode config.	Current (mA)^*^			Pulse width (μs)	Freq. (Hz)	Time to initial monopolar review (days)	Model	Distance to CTT (mm)	Baseline FTM^3^	Post-op FTM	Time to tremor rating score (months)
			Post-op	1-year	2-year								
60	F	Case+,1ABC	2.0	3	3.25	60	130	36	Abbott^1^	4.25	19	7	6
56	M	Case+,3A	1.5	2	3	60	130	29	Abbott^1^	1.59	20	4	2
		Case+,3A	2.5	2.5	5.8	30	180	31	Abbott^1^	4.71	22	8	40
72	M	Case+, 2ABC	1.5	2.5	2.5	60	130	24	Abbott^1^	2.54	8	0	41
		Case+,2ABC	2.0	2.5	2.25	60	130	41	Abbott^1^	4.08	15	2	35
59	M	Case+,2A	0.5	1.5	2.5	60	180	36	Abbott^1^	0.32	15	1	1
		Case+,2ABC	1.2	2.2	3.7	60	180	29	Abbott^1^	3.10	18	1	1
64	F	Case+,1ABC	2.5	3.3	3.7	60	130	31	Abbott^1^	2.99	17	6	3
		Case+,3ABC	2.0	3.5	3.5	30	130	70	Abbott^1^	2.89	11	4	19
65	F	Case+, 1	1.73	2.48	2.48	60	185	53	Medtronic^2^	3.12	11	4	52
69	M	Case+,2ABC	1.7	1.8	1.8	60	130	28	Abbott^1^	4.40	12	2	34
		Case+,3ABC	2.5	2.5	2.5	60	130	28	Abbott^1^	3.41	10	1	33
63.9 ± 5.7			1.8 ± 0.6	2.5 ± 0.6	2.9 ± 1.1			36.3 ± 13.1		3.1 ± 1.2			22.2 ± 18.9

^*^Voltage converted to mA using charted impedances from clinical visit. ^1^Abbott Deep Brain Stimulator Electrode, Infinity 0.5. ^2^Medtronic DBS electrode model #3389. ^3^Fahn-Talosa-Marin hemi-score.

### 2.2. 7T MR imaging acquisition and active contract extraction

Pre-operative ultra-high-field, MR imaging was collected from each subject at the University of Minnesota Center for Magnetic Resonance Research using a 7T research scanner (Magnetom 7T Siemens) and following our published protocol (17, 18). Imaging included 0.6 mm isotropic T1-weighted, 0.4 × 0.4 × 1.0 mm T2-weighted, and 1.25- or 1.5-mm isotropic diffusion-weighted (b = 1,500, 54 directions) images. Subjects returned one-month post-implant for initial programming by a movement disorders specialist. At that time, a post-implant CT scan (0.4 × 0.4 × 0.6 mm) was acquired.

Each subject’s postoperative CT scan was non-linearly coregistered with their preoperative MRI data using elastix (19) to determine the implanted lead location and orientation. The trajectory of the implanted DBS lead shaft is visible on the CT images as hyperintense voxels; the location of the contacts along that trajectory corresponds to hyperintense bulges at the most inferior parts of the trajectory. These can be segmented using a threshold-based segmentation technique and 3D STL files representing the DBS lead elements to scale (shaft + contacts) are placed to fit within the segmentation and bulges. The orientation of the DBS lead and relative direction of individual segments were derived from the fiducial marker on the lead, in combination with the unique artifact characteristics of the segments, using a modified version of the DiODe algorithm (20, 21).

MR images were used to segment nuclei in Slicer (v4.8.0) (22) to be used as waypoints for the CTT. The contralateral deep cerebellar nuclei, the decussation of the superior cerebellar peduncle, and the ipsilateral red nucleus were segmented using the T1- and T2-MR images. Segmentation of the VIM of the thalamus was completed by resampling the T1-MR image to anterior commissure-posterior commissure (AC-PC) space and warping the Mai Atlas of the Human Brain to the T1-MRI images using fiducials placed within and on the thalamic border based on nuclei-specific contrast within the thalamus (10, 11, 23, 24).

### 2.3. CTT pathway modeling

**Figure 1:**
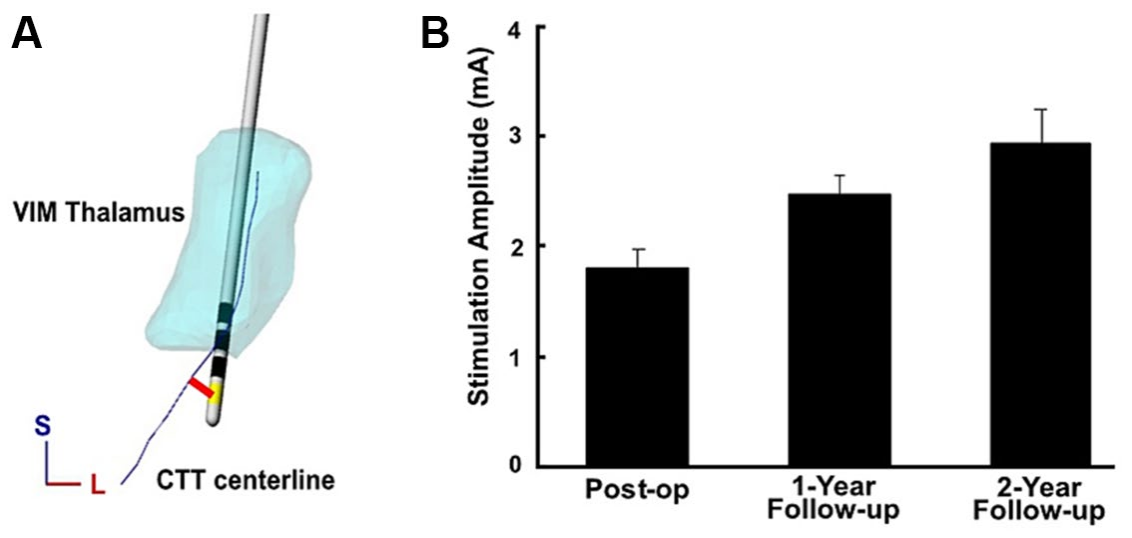
**(A)** The CTT centerline, denoted in blue, was reconstructed from 7T MRI-derived tractography. The Euclidean distance between the center of the active electrode contact (yellow) and the CTT centerline was then calculated (red). An example is shown from a single subject. **(B)** Therapeutic current at initial post-op programming as well as at 1-year and 2-year follow ups across implants, with errors bars displaying the standard error.

Modeling of the CTT was completed using diffusion-weighted images. These images were first processed with FMRIB’s Software Library (FSL) using the following tools: FDT was used for eddy-current correction, BEDPOSTX was used to estimate the diffusion parameters, and DTIFIT was used to fit the diffusion model to each voxel (FMRIB, Oxford, United Kingdom) (25–28). Probabilistic tractography was then completed to extract subject-specific trajectories for the CTT. Seed-points were placed in the deep cerebellar nuclei (DCN) contralateral to the DBS lead, waypoints were placed in the decussation of the superior cerebellar peduncle and the ipsilateral red nucleus/lateral edge of the red nucleus, and endpoints were placed in the ipsilateral VIM of thalamus. Segmentations of the CTT and reconstructions of the active electrode(s) on each DBS lead were imported into Rhinoceros 3D (v4.0; Robert McNeel & Associates, Seattle, WA, United States) to create a patient specific volume models as seen in Figure 1A. A skeletonization algorithm designed in MATLAB was used to calculate the centerline of the segmented CTT volume and the center point of the active electrode(s). These data were then exported to MATLAB (R2019b; The MathWorks, Inc., United States), where a script was used to calculate the distance from the active electrode center point to the closest point on the CTT centerline.

### 2.4. Statistical analysis

The primary analysis utilized univariate linear regression with stimulation amplitude as a dependent variable and distance to CTT serving as an independent variable. A secondary analysis was performed with clinical tremor control as a dependent variable, and distance to CTT serving as the independent variable. Clinical tremor control was calculated in two ways, the first calculated the total improvement in the FTM hemi-score, subtracting the post-operative score from the baseline score. A second method calculated the percentage reduction in tremor. A multivariable regression model was utilized to test the hypothesis that clinical tremor control was predicted by initial stimulation amplitude when adjusted for age, baseline disease severity, and distance to CTT. We also tested the hypothesis that clinical tremor control was predicted by distance to CTT using univariable regression. Summary statistics with normally distributed continuous variables were presented as means and (SD). Student’s t-test was used to test differences in means. In all tests, significance was set at *p* < 0.05.

## 3. Results

**Figure 2:**
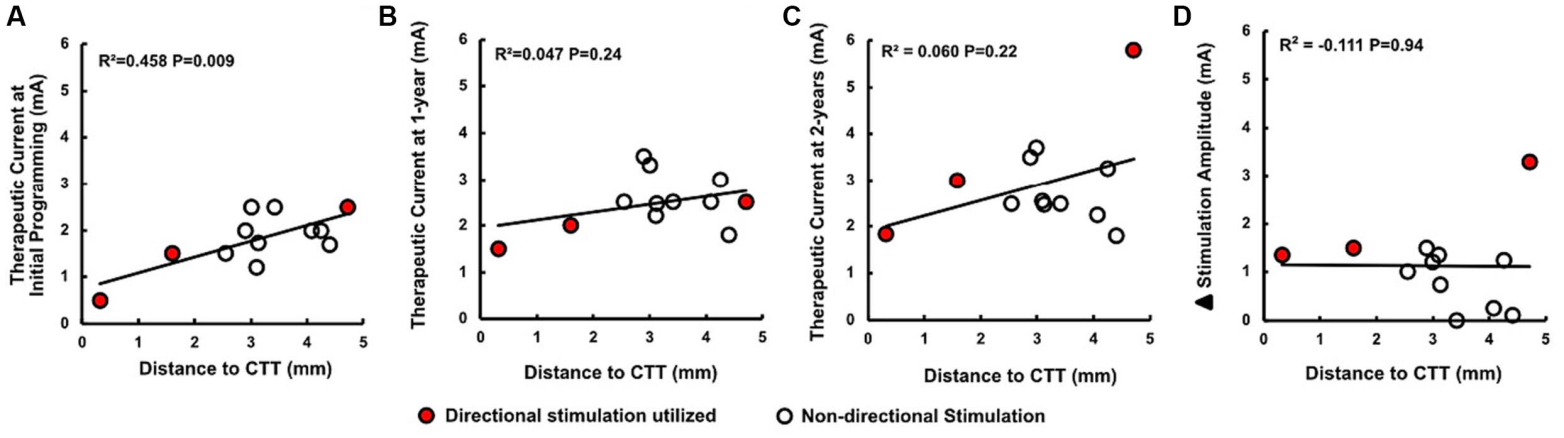
**(A–C)** Active contact proximity to the CTT was associated with lower therapeutic current at the time of initial programming; however, this association was not found at the 1-year and 2-year follow ups. **(D)** Distance to CTT did not predict 𝚫 stimulation amplitude, defined as the difference between therapeutic current at initial and 2-year follow up. Data points demarcated in red symbolize the use of a directional (segmented) electrode.

The mean (SD) age of subjects was 63.9 (±5.7) years, and time to monopolar review and initial programming after implantation was 36.3 (±13.1) days (Supplementary Table 2). Mean (SD) distance from the active contact to the CTT was 3.1 mm (±1.2; Figure 1A). Therapeutic current was 1.8 mA (±1.8) at initial programming, then subsequently increased to 2.5 mA (±0.6) at 1-year and 2.9 mA (±1.1) at the 2-year follow up (Figure 1B). Active contact proximity to the CTT was associated with lower therapeutic current at the time of initial programming (R^2^ = 0.458, *p* = 0.009); however, this association was not significant at the 1-year and 2-year follow up (Figure 2). Distance to the CTT did not predict the need for an increase in therapeutic current over time. Persistent side effects evoked with supratherapeutic stimulation were as follows: 7 patients experienced dysarthria, 2 patients with contralateral hand numbness, 1 patient experienced ataxia, 1 patient facial numbness, and 1 patient with an out of body experience. Mean (SD) stimulation threshold for evoking persistent side effect was 2.9 mA (±0.8). Distance to CTT did not predict the side effect phenotype or the stimulation threshold (R^2^ = 0.043, *p* = 0.24) for induction of side effects observed at the initial programming session.

**Figure 3:**
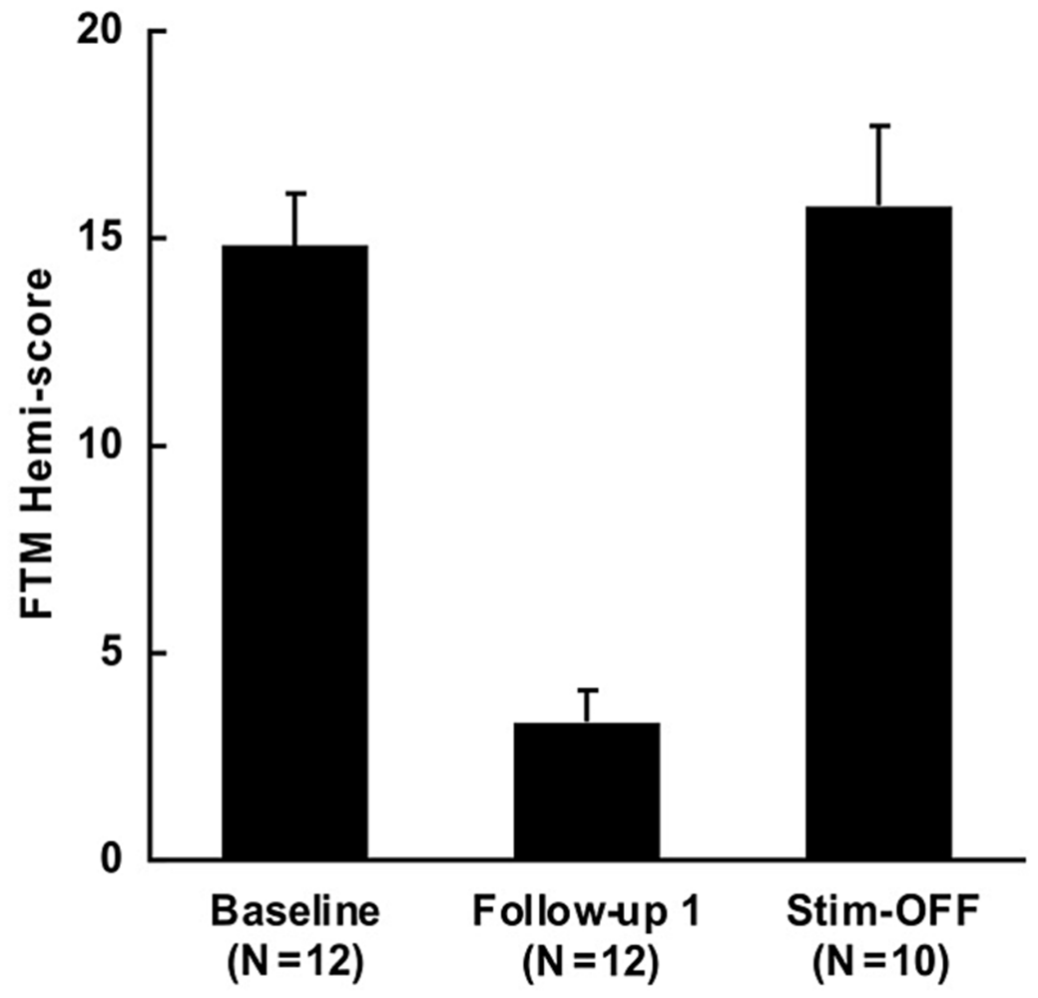
Improvement in FTM hemi-score with VIM DBS for ET. Baseline FTM hemi-score was 14.8 (±4.4), with post-operative score of 3.3 (±2.6) at mean follow up of 22.2 (±18.9) months. Mean OFF-stim score was 15.8 (±6.0) at mean follow up of 26.1 (1.7) months. FTM hemi-score values are represented as means, error bars as standard errors.

**Figure 4:**
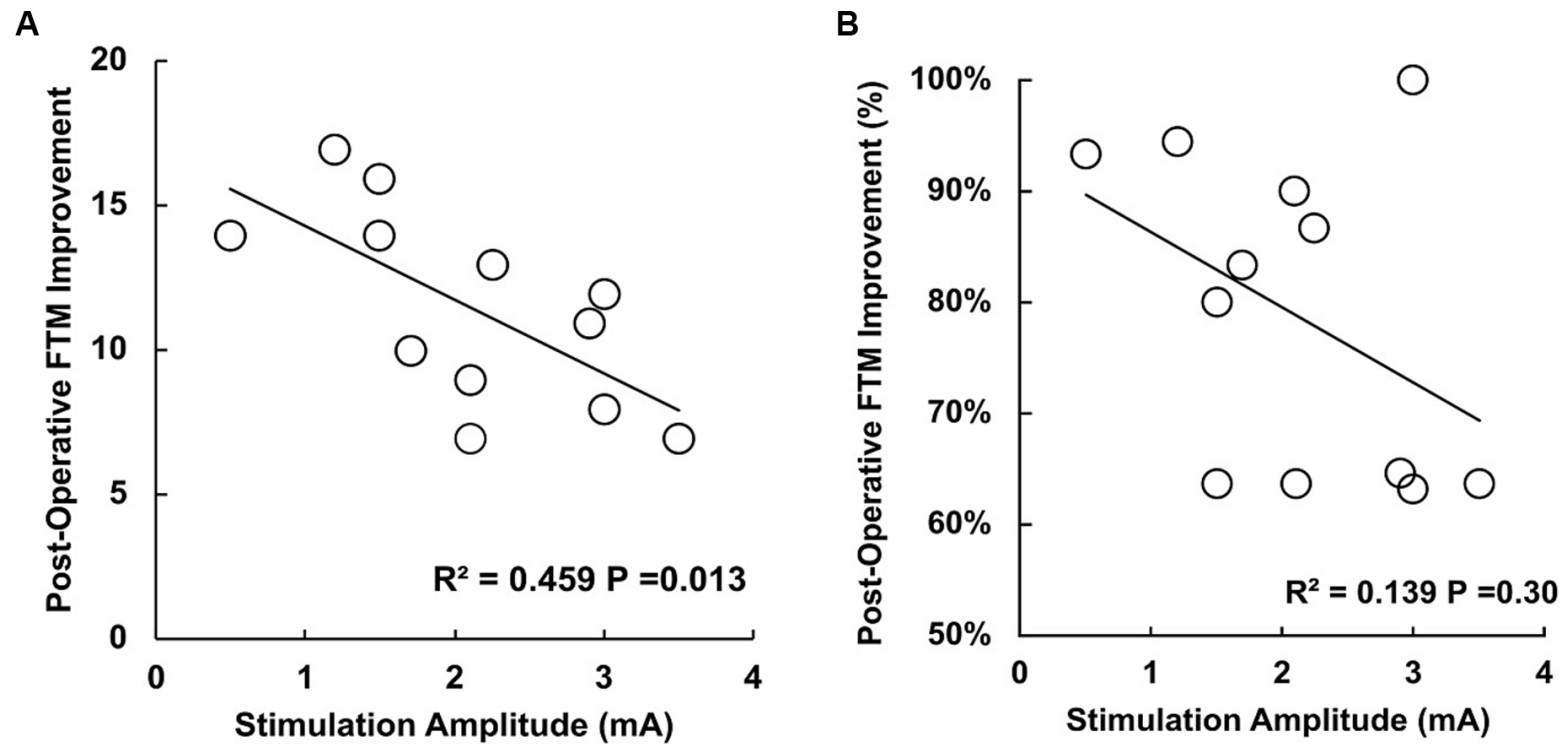
**(A)** Scatter plot with improvement in the FTM hemi-score after surgery (mean time to collection 22.2 months) in the Y axis and the stimulation applied in the X axis. Improvement in FTM score is defined as the difference between the baseline FTM hemi-score and post-operative FTM hemi-score. Subjects with lower stimulation amplitudes at the initial programming had better post-operative tremor control (R^2^ = 0.459, *p* = 0.013). **(B)** Scatter plot with post-operative FTM hemi-score displayed as a percentage improvement from baseline FTM hemi-score. In this context, the finding was not statistically significant (R^2^ = 0.139, *p* = 0.30).

The mean (SD) pre-operative FTM hemi-score was 14.8 (±4.4) and post-operative FTM hemi-score was 3.3 (±2.6) at mean follow up of 22.2 (±18.9) months. This represented a mean (SD) improvement of 77.9% (±14.5%) when scaled to baseline. To assess the degree of tremor without stimulation post-implant, OFF-stim scores were collected in 10 patients, with a mean of 15.8 (±6.0) as seen in Figure 3. Mean time to collection of these OFF-stim scores was 26.1 (1.7) months. Subjects with lower stimulation amplitudes at the initial programming had better post-operative tremor control (R^2^ = 0.459, *p* = 0.013) in univariable regression (Figure 4A) as well as when adjusting for age, baseline tremor severity, time to follow up, and distance to CTT (R^2^ = 0.845, *p* = 0.0036). However, when plotting post-operative tremor control as a percentage of baseline FTM (1-[post-op FTM-baseline FTM]), this finding was not statistically significant (R^2^ = 0.139, *p* = 0.30) as seen in Figure 4B. Additionally, distance to CTT did not predict post-operative tremor control at the time of clinical follow up (R^2^ = −0.073, *p* = 0.58).

## 4. Discussion

This study sought to characterize the relationship between the proximity of the active DBS contact to the CTT and post-operative stimulation parameters. 7T MRI tractography techniques permit a defined spatial delineation of the CTT, enabling more precise exploration of this relationship. The results in this small cohort of patients suggest that lower therapeutic current is required to suppress tremor when active contacts are situated near the CTT, implying that activation of the CTT plays a role in tremor suppression. However, this relationship weakened at 1- and 2-year follow ups, indicating that over time distance to CTT does not fully explain the observed effects of DBS on CTT. Other factors, such as habituation to stimulation, percentage of CTT axons activated, stimulation field orientation, tissue susceptibility to stimulation, local tissue reactions to the implanted lead (29, 30) tremor phenotype or activation of other critical structures likely also influence the degree of tremor response.

Our data indicate that active contact proximity to CTT is associated with lower therapeutic current following DBS surgery for ET. While CTT is an important pathway in motor control the nature of this relationship, especially in the setting of DBS, is not well understood (31). In a prospective cohort of 7 patients, the stereotactic coordinates of the most effective stimulation contacts were located closer to the CTT, specifically via the dentate nucleus and its projections to thalamus (8). Our results are consistent with this outcome. However, while effectiveness was defined in Groppa et al. by tremor suppression, we found that lower therapeutic currents were required when subjects were clinically optimized at the time of initial programming (Figure 2A). Tremor suppression may be achieved at a lower stimulus amplitude due to the role of the CTT in the production of ET tremor or the tighter grouping of CTT fibers ventral to the VIM, where many of the active electrodes were located. Further study is needed to investigate whether lower therapeutic currents could serve as a biomarker of optimized contact location following DBS lead placement.

A secondary finding in our study is that lower therapeutic current requirements were associated with better tremor outcomes after DBS surgery. However, this was only statistically significant when tremor reduction was calculated as a score reduction in the FTM hemi-score from baseline. Ongoing research is needed, perhaps in a larger cohort, to clarify the relationship between therapeutic current needs and the degree of tremor reduction after surgery. Current requirement is likely to be a variable that reflects a composite of factors, likely including distance to the CTT, that is essential for obtaining the best outcomes. To build on this complexity, we did not find that distance to CTT predicted long-term clinical outcome. This could be due to long follow up times, alterations in the current field effects on axonal membrane properties, or the variable circumstances under which these clinical tremor scores were collected. While the relationship between a low stimulation amplitude and clinical success has long been recognized by DBS practitioners, we are unaware of efforts to quantitatively explore this relationship. Further study is needed to understand the factors that affect CTT activation, as surrounding neuroanatomical structures and their tissue densities may impact the way DBS activates fiber pathways. Activation of the CTT at different points may result in different clinical outcomes or tremor phenotypes, which will need to be elucidated in future studies.

This study contains several limitations. Its small, retrospective design limits its statistical power and may limit its generalizability across a broader cohort of patients. The lack of long-term clinical follow-up data such clinical tremor rating scores impairs our ability to comment on the contribution of habitation to our long-term data. In addition, there is considerable inhomogeneity in the time to clinical follow up, necessitating that the relationships we show here between stimulation amplitude, distance to CTT, and tremor improvement must be interpreted with caution. Our method of determining distance to CTT relied on measuring a Euclidean distance between the active contact and the centerline of the tract, which does not account for nuanced differences in pathway activation that may occur when considering the relative location of the contacts (e.g., medial or lateral) with respect to the CTT. An analysis that determined specifically the degree of pathway activation would be of benefit in future studies and remains a limitation of our approach here. This is especially true in cases where directional stimulation was utilized, as this may affect which pathways are activated subsequently changing the tremor reduction and side effect thresholds measured in our data set. In the 3 cases here which utilized directional stimulation, 2 were directed at CTT, another was deviated further from the CTT centerline. Nordin et al. has previously utilized finite element method combined with tractography to better delineate the boundaries of activated tissue and if pathways of interest fall within it. It may therefore represent a path forward in integrating these analyses into clinical practice (32). Our modeling methodology did not consider the width of the CTT, as all calculations measured the difference from the centerline of the tract to the active electrode contact. It is also worth noting that segmented CTT reconstruction volume can depend on the thresholds used in the probabilistic tractography calculation; however, the tractography approach was guided by visible fiducials (e.g., red nucleus) from the 7T MR T1- and T2-weighted imaging providing a higher degree of confidence in the CTT centerline calculation.

In conclusion, VIM DBS can be a highly effective procedure for achieving tremor control in medically refractory ET. However, clinical outcomes can vary significantly across and within centers. There is a clinical need to better define the variables that predict outcomes after surgery and relate these to DBS mechanisms in ET. In our patient cohort, subjects with active contact locations near the CTT had lower therapeutic current requirements following VIM DBS surgery for ET. However, distance to CTT did not predict the long-term clinical outcome. This finding further reinforces the importance of further research into the relationship between cerebellothalamic pathways and DBS in ET patients.

## Data availability statement

The original contributions presented in the study are included in the article/Supplementary material, further inquiries can be directed to the corresponding author.

## Ethics statement

The studies involving humans were approved by the University of Minnesota Institutional Review Board. The studies were conducted in accordance with the local legislation and institutional requirements. The participants provided their written informed consent to participate in this study.

## Author contributions

SI: Conceptualization, Data curation, Formal Analysis, Writing – original draft, Writing – review & editing. AB: Writing – original draft, Writing – review & editing. RB: Data curation, Writing – review & editing. MH: Formal Analysis, Writing – review & editing. RD: Writing – review & editing. JA: Methodology, Writing – review & editing. LS: Writing – review & editing. SC: Methodology, Writing – review & editing. TP: Data curation, Writing – review & editing. RP: Data curation, Writing – review & editing. NH: Data curation, Writing – review & editing. JV: Conceptualization, Data curation, Formal Analysis, Funding acquisition, Investigation, Methodology, Project administration, Resources, Supervision, Writing – original draft, Writing – review & editing. MJ: Conceptualization, Data curation, Formal Analysis, Funding acquisition, Investigation, Methodology, Project administration, Resources, Software, Supervision, Validation, Visualization, Writing – original draft, Writing – review & editing.
