# Supplementary material for: Active contact proximity to the cerebellothalamic tract predicts initial therapeutic current requirement with DBS for ET: an application of 7T MRI
Source: Front Neurol. 2023 Nov 9;14:1258895. doi: 10.3389/fneur.2023.1258895 (PMC10666159; doi:10.3389/fneur.2023.1258895)
Supplement: Supplementary file 1 [file Table_1.DOCX]

**Supplementary Table S1: Fahn-Talosa-Marin Hemi-score (36 max)**

| **Clinical Assessment** | **Description** | **Scoring** |
| --- | --- | --- |
| Resting Tremor (upper/lower extremity) | Tremor at rest while supine | 0 = None  1 = Amplitude < 0.5 cm  2 = Amplitude 0.5-1.0 cm  3 = Amplitude 1-2 cm  4 = Amplitude > 2 cm |
| Postural Tremor (upper/lower extremity) | UE: Arms outstretched, wrists mildly extended, fingers spread  LE: Legs flexed at hips/knees; foot dorsiflexed |  |
| Action Tremor (upper/lower extremity) | UE: Finger-to-nose  LE: Toe-to-Finger |  |
| Handwriting | “This is a sample of my best handwriting” | 0 = Normal  1 = Untidy, tremulous  2 = Legible  3 = Illegible  4 = Unable to provide sample |
| Drawing A | Large spiral | 0 = Normal  1 = May cross lines occasionally  2 = Crosses lines frequently  3 = Many errors  4 = Unable to complete drawing |
| Drawing B | Small spiral |  |
| Drawing C | Straight lines |  |
| Pouring Liquids | Using an 8cm cup, filled 1cm from the brim, pour water from one cup to another. | 0 = Normal  1 = Poured carefully, no spills  2 = Spills <10% of water volume  3 = Spills 10-50% of water volume  4 = Unable to pour water |
